# The Challenging Diagnosis of Primordial Odontogenic Tumor

**DOI:** 10.1155/2019/6415785

**Published:** 2019-04-24

**Authors:** Lucas Novaes Teixeira, Cristiane Furuse, Fabrício Passador Santos, Andresa Borges Soares, Eder Magno Ferreira de Oliveira, Ney Soares de Araújo, Vera Cavalcanti de Araújo

**Affiliations:** ^1^Faculdade São Leopoldo Mandic, Campinas, SP, Brazil; ^2^School of Dentistry, São Paulo State University, Department of Pathology and Clinical Propedeutics, Araçatuba, SP, Brazil; ^3^Oral and Maxillofacial Surgery, Dr. Mário Gatti Municipal Hospital, Campinas, SP, Brazil

## Abstract

Primordial odontogenic tumor (POT) is a benign mixed odontogenic tumor comprised of a loose connective tissue with a similar morphology with dental papilla and exhibiting in its periphery the presence of a columnar epithelium. POT occurs in young patients and typically is associated with an unerupted tooth, with the mandible being the main anatomic site of occurrence. The present manuscript is aimed at describing a new case of POT and reviewing the main biologic findings related to this odontogenic tumor.

## 1. Introduction

The classification of lesions derived from the epithelium of the odontogenic apparatus or from its derivatives or remnants has always been challenging for the pathologists. This difficulty can be noticed through the different editions of *World Health Organization (WHO) Classification of Head and Neck Tumours*. Overall, the classification of these lesions, irrespective of the WHO editions, is based on biological behavior (benign and malignant) and histologic origin, i.e., epithelial, epithelial with ectomesenchyme (mixed), and ectomesenchymal tumors [1]. In the 4th edition of WHO, besides the return of the odontogenic cysts, two new entities were included: sclerosing odontogenic carcinoma and primordial odontogenic tumor [2, 3].

Primordial odontogenic tumor (POT) was firstly described by Mosqueda-Taylor et al. [4], being classified as a benign mixed epithelial and mesenchymal odontogenic tumor in the current *WHO Classification of Head and Neck Tumours* [3]. In English language literature, there are fourteen described cases of POT occurring predominantly in young patients in the first and second decades, with the mandible being the most prevalent site. There is a slight predilection for males, and the lesions are usually associated with an unerupted tooth [4–12]. In the present manuscript, we aimed to report a new case of POT and review the main biological findings with regard to the nature of this tumor.

## 2. Case Report

**Figure 1:**
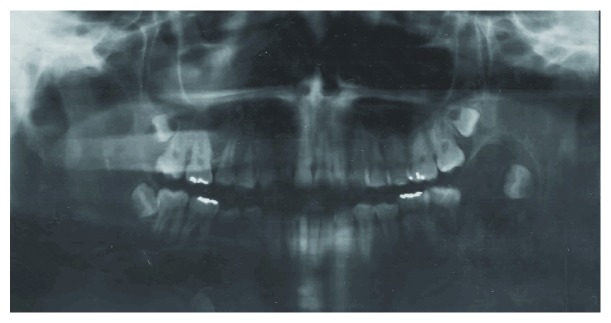
Radiolucent and well-delimited lesion surrounding the third molar in the left side of the mandible.

In 2006, a 13-year-old black female presented to Dr. Mário Gatti Municipal Hospital with a complaint about a volume augmentation on the left side of her mandible for 3 months. Her medical history was not contributory. Panoramic radiography revealed a well-delimited radiolucent lesion circumscribing the tooth germ of the third molar (Figure 1). The clinical suspicion was dentigerous cyst, odontogenic keratocyst, or ameloblastoma.

Two incisional biopsies followed by an excisional biopsy were performed, and the specimens were fixed in 10% buffered formalin. Paraffin sections were prepared for light microscopy using routine procedures. The sections were stained with hematoxylin and eosin. At that time, the first histologic diagnosis for incisional biopsy was dental papilla, while the histologic diagnoses for the other biopsies were inconclusive and compatible with a developing tooth. In 2014, the description of new odontogenic entity called POT leads us to revise the present case, which exhibited histologic similarities with the cases described by Mosqueda-Taylor et al. [4].

**Figure 2:**
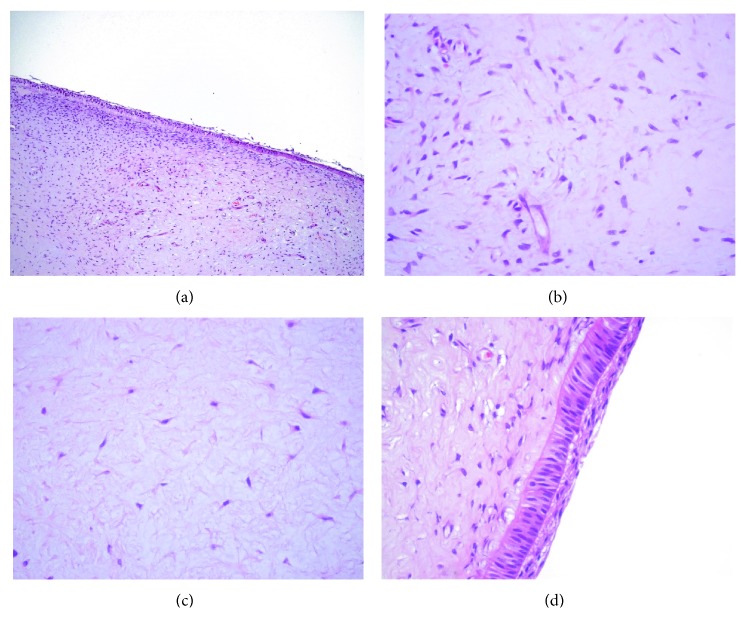
Microscopically, the lesion was characterized by a loose connective tissue recovered by a columnar epithelium (a). Areas with different cell densities were noticed in the specimen (b, c). In some regions, upon the columnar cells, 3 layers of stratified squamous cells were detected exhibiting an outer enamel epithelium-like morphology (d). Scale bar: (a): 80 *μ*m; (b–d): 20 *μ*m.

Histologic analysis revealed a fragment of loose connective tissue covered with the epithelium exhibiting predominantly a columnar morphology (Figure 2(a)). In the connective tissue, areas with a great number of cells showing a morphology ranging from fusiform to stellate morphology were noticed (Figure 2(b)). On the other hand, regions with low cell density and myxoid appearance were also observed in the connective tissue (Figure 2(c)). The epithelium was characterized by the presence of columnar cells. In some areas, the columnar cells were covered by a stratified squamous epithelium, which was interpreted as similar to the outer enamel epithelium of the enamel organ (Figure 2(d)). Calcified areas and/or odontogenic epithelial islands or cords were not detected in any part of the specimen. These histologic findings rendered the diagnosis of POT.

## 3. Discussion

POT was first described by Mosqueda-Taylor et al. In their series of six cases, these authors reported a new odontogenic lesion that did not fit in any category of odontogenic tumors described before [4, 13]. After the establishment of this new pathologic entity, eight cases were reported in English language literature [5–12]. The majority of the cases were diagnosed in young patients, with the mandible being the main anatomic site of occurrence. All cases reported were asymptomatic and presented an expansion of cortical bone. Radiographically, the lesions exhibited a large and well-defined radiolucency involving completely an unerupted tooth, particularly the third molar. The prognosis of this tumor is good, and no recurrence was described. Our case fulfills all clinical and radiographic criteria of POT, as described above, and until now no recurrence was reported.

Concerning the histological features, POT is characterized by variably cellular to loose fibrous tissue with areas mimicking dental papilla, being circumscribed by epithelial cells showing morphologies ranging from cuboidal to columnar [4]. In our case, these microscopical findings were also observed as demonstrated in the images present in the manuscript. These histological findings suggested that this new odontogenic lesion was probably derived from a tissue very similar to dental papilla or even from an abortive tooth germ, which leads Mosqueda-Taylor et al. to coin the term “primordial odontogenic tumor” [4]. Interestingly, none of the cases of POT described in the literature reported the absence of a tooth in the tumor area.

Several studies have been conducted to comprehend the etiopathogenesis of POT. Bologna-Molina et al. carried out a vast immunohistochemistry panel for several markers, such as cytoskeleton proteins, endothelial surface receptors, extracellular matrix proteins, and proliferation and apoptotic markers [14]. The results obtained by these authors lead them to conclude that POT is a slow-growing and moderately vascularized tumor with variable secretion of loose fibrous tissue [14]. Immunohistochemistry analysis conducted by Mikami et al. revealed the presence of vimentin- and smooth muscle actin-positive cells in the fibrous connective tissue and the expression of cytokeratins 14 and 19 in the surrounding epithelium, with the latter being detected chiefly in cuboidal/columnar epithelial cells [7], which is consistent with morphological differentiation towards the ameloblastic phenotype [15]. Interestingly, all peripheral epithelium layers showed moderate vimentin positivity, and cytokeratin 18 was positive only in the inner enamel epithelium-like epithelium. This pattern of vimentin and cytokeratin immunoexpression is also observed during tooth development, which reinforces the hypothesis that POT is derived from a primordial tooth germ [7]. Besides, the similar syndecan 1 and Ki-67 profile expression between POT and normal tooth germs [10], as well as the presence of the transcription factor PITX2 in focal areas of the POT epithelium, also supports the theory that this tumor probably derived from early stages of tooth development [14].

In mixed odontogenic tumors, mineralized tissues, such as dentin and enamel, can be present due to the reciprocal inductions between epithelial and mesenchymal cells. The presence of mineralized material, however, is not a common histological feature described in POT. Mikami et al. did not detect somatic mutations in any amelogenesis- and dentinogenesis-related genes by DNA analysis. The mRNA levels of some dentinogenesis-associated genes, however, were significantly variable. Collagen type I and dentin sialophosphoprotein (DSP) mRNA were highly expressed in POT, suggesting the presence of preodontoblasts in this tumor. However, the presence of cells exhibiting a morphology compatible with a fully differentiated odontoblast was not observed [16]. In accordance with these findings, in our case, we detected some areas with high mesenchymal cell density beneath the epithelium, but we did not find any cell with a morphology similar to that of an odontoblast. This fact can be attributed to the disturbance of the process of translation of the DSP gene to DSP, dentin glycoprotein, and dentin phosphoprotein, essential proteins for the normal odontoblastic differentiation [16].

In conclusion, this case report brings an important message to pathologists to prevent misdiagnosis. An incisional biopsy may carry only the mesenchymal component of POT, which constitutes the main bulk of this tumor and, consequently, may lead to misdiagnosis, such as dental papilla or even odontogenic myxoma. The analysis of the total area of this tumor, associated with careful interpretation of radiographic images is paramount for correct diagnosis of this tumor.
